# Transcriptomic Analysis Reveals Insights on Male Infertility in *Octopus maya* Under Chronic Thermal Stress

**DOI:** 10.3389/fphys.2018.01920

**Published:** 2019-01-15

**Authors:** Laura López-Galindo, Oscar E. Juárez, Ernesto Larios-Soriano, Giulia Del Vecchio, Claudia Ventura-López, Asunción Lago-Lestón, Clara Galindo-Sánchez

**Affiliations:** ^1^Laboratorio de Genómica Funcional, Departamento de Biotecnología Marina, Centro de Investigación Científica y de Educación Superior de Ensenada, Ensenada, Mexico; ^2^Laboratorio de Fisiología Integrativa de Organismos Marinos, Departamento de Biotecnología Marina, Centro de Investigación Científica y de Educación Superior de Ensenada, Ensenada, Mexico; ^3^Facultad de Ciencias, Universidad Nacional Autónoma de México, Mexico City, Mexico; ^4^Departamento de Innovación Biomédica, Centro de Investigación Científica y de Educación Superior de Ensenada, Ensenada, Mexico

**Keywords:** male infertility, chronic thermal stress, RNA-Seq, reproduction, inflammation

## Abstract

*Octopus maya* endemic to the Yucatan Peninsula, Mexico, is an ectotherm organism particularly temperature-sensitive. Studies in *O. maya* females show that temperatures above 27°C reduce the number of eggs per spawn, fertilization rate and the viability of embryos. High temperatures also reduce the male reproductive performance and success. However, the molecular mechanisms are still unknown. The transcriptomic profiles of testes from thermally stressed (30°C) and not stressed (24°C) adult male octopuses were compared, before and after mating to understand the molecular bases involved in the low reproductive performance at high temperature. The testis paired-end cDNA libraries were sequenced using the Illumina MiSeq platform. Then, the transcriptome was assembled *de novo* using Trinity software. A total of 53,214,611 high-quality paired reads were used to reconstruct 85,249 transcripts and 77,661 unigenes with an N50 of 889 bp length. Later, 13,154 transcripts were annotated implementing Blastx searches in the UniProt database. Differential expression analysis revealed 1,881 transcripts with significant difference among treatments. Functional annotation and pathway mapping of differential expressed transcripts revealed significant enrichment for biological processes involved in spermatogenesis, gamete generation, germ cell development, spermatid development and differentiation, response to stress, inflammatory response and apoptosis. Remarkably, the transcripts encoding genes such as ZMYND15, KLHL10, TDRD1, TSSK2 and DNAJB13, which are linked to male infertility in other species, were differentially expressed among the treatments. The expression levels of these key genes, involved in sperm motility and spermatogenesis were validated by quantitative real-time PCR. The results suggest that the reduction in male fertility at high temperature can be related to alterations in spermatozoa development and motility.

## Introduction

*Octopus maya* endemic to the Yucatan Peninsula (YP) is an ectotherm organism particularly temperature-sensitive mainly due to the characteristics of its habitat (Noyola et al., 2013a,b; Regil et al., 2015). It is one of the most important commercial fisheries in the YP and the American continent (Gamboa-Álvarez et al., 2015). The YP is divided into two distinct zones. The eastern zone located in front of the Yucatan state presents a summer upwelling that brings a mass of cold water from the Caribbean (16–22°C) that enters the YP and acts as an external temperature control for the shelf with temperatures fluctuating between 23 and 27.5°C (Noyola et al., 2013b). Meanwhile, the western zone located in front of Campeche has no influences of deep cold waters, and as a consequence, the surficial temperatures can rise above 30°C in summer (Noyola et al., 2013b; Regil et al., 2015). Gamboa-Álvarez et al. (2015) observed that temperature modules two essential aspects in the YP such as the fishing seasons which presents higher octopus abundances with low biomass in the western zone. In the eastern zone, lower abundances and higher biomass had been recorded. In the other hand, low temperatures in the different zones of the YP favor the spawning (Avila-Poveda et al., 2015; Gamboa-Álvarez et al., 2015; Regil et al., 2015).

Temperature plays a crucial role in different life aspects of *O. maya*. In females, it has been observed that temperatures above 27°C inhibit the spawning and drastically reduce the eggs production, the fertilization rate, the embryonic development time, the number of hatchlings and hatchling survival (Juárez et al., 2015, 2016). In embryos, high temperatures increase the metabolic rates affecting the embryo development (smaller embryos) and hatching rate. Embryos have a thermal threshold at 26°C and temperatures around 30°C inhibit growth, reduce the metabolic rate and embryos present a high yolk proportion (Caamal-Monsreal et al., 2016; Sanchez-García et al., 2017). Recently, in males of *O. tankahkeei*, through histological analysis in the testis of octopus exposed to 32°C for 2 h, Long et al. (2015) observed ultrastructural changes and damaged mitochondria in spermatocytes and spermatids.

The testis is the male gonad, responsible for the production of male gametes via spermatogenesis and androgenic hormones (Waiho et al., 2017). The spermatogenesis is a dynamic, synchronized and highly regulated process that involves the division and differentiation of spermatogonial germ cells into mature spermatozoa, which take place in the seminiferous tubules (Shaha et al., 2010; Akmal et al., 2016). The normal process begins with a spermatogenic phase regulated by mitotic divisions, followed by two meiotic divisions to produce secondary spermatocytes and ends with spermiogenesis, a remarkably morphological transformation process. The spermiogenesis involves: (a) nucleus condensation, where DNA is compacted by protamines; (b) formation of acrosome that contains hydrolytic enzymes crucial for oocyte penetration during fertilization; (c) flagellum formation, which involves the development of microtubules arising from the centrioles of the round spermatid; and (d) cytoplasm reorganization, where a large part of the cytoplasm is phagocytosed by the Sertoli cells, that constitutes a hematotesticular barrier (Sheng et al., 2014).

The morphology and ultrastructure of testis and germ cells in *O. maya* and their histological changes during sexual maturation has been described in detail by Avila-Poveda et al. (2009, 2016). In a previous work of our team, the effect of thermal stress over the physiology and the reproductive performance and success of male *O. maya* exposed to preferred (24°C) and stress (28 and 30°C) temperatures was assessed. Our research findings indicated that chronic thermal stress inhibited the growth rate: organisms exposed to 30°C had a specific growth rate six times lower than those exposed to 24°C and gained weight nine times lower. A significant reduction in oxygen consumption was identified with increasing temperatures. High temperatures induced the immune response in *O. maya* males by increasing the circulating hemocytes in the hemolymph. At the reproductive level, a significant increment in the production of spermatophores with increasing temperatures was observed. Although, despite this reproductive strategy, the reproductive success was affected, with no parental contribution from octopus exposed to 30°C. The histological analysis of the testis showed damage from moderate to severe in octopus exposed to 28 and 30°C, seriously affecting the cellular testis organization (López-Galindo et al., 2019).

Nevertheless, the molecular mechanisms that regulate reproduction process in *O. maya* males and the response to environmental factors as temperature are poorly understood. The transcriptome analysis through RNA-Seq methodology could reveal transcripts that are being actively expressed in testis of *O. maya* under chronic thermal stress and facilitate the discovery of novel genes involved in this response and the reproductive processes with high sensitivity and accuracy as has been successfully identified in other invertebrate species as the Pacific oyster *Crassostrea gigas* (Lim et al., 2016; Kim et al., 2017), green lip abalone *Haliotis laevigata* (Shiel et al., 2014), snail *Echinolittorina malaccana* (Wang et al., 2014), king scallop *Pecten maximus* (Artigaud et al., 2015), Chinese mitten crab *Eriocheir sinensis* (Li and Qian, 2017), orange mud crab *Scylla olivacea* (Waiho et al., 2017), and squid *Loligo bleekeri* (Yoshida et al., 2014). To date, in cephalopods transcriptome information related to reproduction is still insufficient. In this regard, this study aims to provide insights into the molecular mechanisms that regulate the reproductive processes such as spermatogenesis and spermiogenesis in testis of *O. maya* under chronic thermal stress. Here, we present a comprehensive analysis of the transcriptome data obtained from testis tissue of *O. maya* males exposed to optimal, intermediate and stressful temperature before and after mating using Illumina Miseq. This is the first report of how octopus male fertility is affected at the molecular level and which mechanisms are triggered to cope with chronic thermal stress. Our results indicated that despite the adaptative mechanisms present in *O. maya* to tolerate temperatures close to 30°C, apparently a prolonged exposure to them causes infertility related to alterations in sperm development and motility.

## Materials and Methods

### Ethics Statement

We established protocols that were approved by the Experimental Animal Ethics Committee of the Faculty of Chemistry at Universidad Nacional Autónoma de México (Permit No. Oficio/FQ/CICUAL/099/15). Octopuses were anesthetized with 3% ethanol to induce narcotization to enable humane killing in consideration of animal’s welfare during manipulations (Mather and Anderson, 2007; Estefanell et al., 2011; Andrews et al., 2013; Gleadall, 2013).

### Experimental Design and Sampling

*Octopus maya* males were captured off the coast of Sisal Yucatan, from June to September of 2015, we obtained a total of 63 testis samples. Thirty-six testis were sampled from males before the copula (PRE) that were maintained in 80 L individual tanks and exposed at three experimental temperatures during 30 days (*n* = 12 per treatment): (a) preferred temperature (24°C; 24PRE); (b) intermediate temperature (28°C; 28PRE); and (c) stress temperature (30°C; 30PRE). Meanwhile, twenty-seven testis were sampled from males after the copula (POS), exposed to chronic thermal stress and mated with females maintained at 24°C (*n* = 9 per temperature). Testis samples were removed surgically and immediately preserved in Nap buffer (Camacho-Sanchez et al., 2013), and stored at -80°C until required. Furthers details are shown in López-Galindo et al. (2019).

### RNA Isolation, Library Preparation, and Sequencing

Total RNA was obtained from 30 mg of testis tissue homogenized in Fastprep-24 Instrument (MP Biomedicals, Solon, OH, United States) with a speed of 5.0 m/s for 20 s. Then, total RNA was extracted using the RNEasy Plus mini kit (Qiagen, Hilden, Germany) following the manufacturer’s protocol. Total RNA samples were then digested with RQ1 RNase-Free DNase (Promega, Madison, WI, United States) to remove potential genomic DNA contamination using the manufacturer’s protocol with an additional precipitation and purification steps as follows: each treated sample was precipitated with 1:10 volumes of 3 M sodium acetate and three volumes of absolute Ethanol at -80°C for 1 h; centrifuged at 13,000 rpm for 10 min at 4°C. The RNA pellets were washed with 200 μl of cold 70% Ethanol; centrifuged at 7,500 rpm for 10 min at 4°C and dried at room temperature for 10 min. The RNA pellets were resuspended in RNase free-DNase water. The quality of the RNA was assessed by 1% agarose gel electrophoresis and quantified using a Nanodrop 2000 spectrophotometer (Thermo Scientific, Wilmington, DE, United States). For transcriptomic analysis, only the organisms exposed to 24 and 30°C were sequenced. To construct the libraries, we prepared three different pools with equal amounts of RNA from four individuals per experimental condition (24PRE and 30PRE, 24POST and 30POST).

The quality of the 12 RNA pools (three pools 24PRE, three pools 24POST, three pools 30PRE, and three pools 30POST) were analyzed with an Agilent 2100 Bioanalyzer system (Agilent Technologies, Santa Clara, CA, United States). cDNA libraries were prepared using the TruSeq^®^RNA Sample Prep kit V2 (Illumina, San Diego, CA, United States) following manufacturer’s protocol. Amplified libraries were purified with AMPure XP magnetic beads (Beckman Coulter, Brea, CA, United States). The fragment sizes were verified and quantified with the 2100 Bioanalyzer system. The 12 paired-end libraries were normalized at 4 nM and then pooled equally. They were sequenced using the MiSeq Reagent Kit v3, with a read length of 2 bp × 75 bp on Illumina MiSeq sequencing system (San Diego, CA, United States). PhiX control was used at 1% for cluster generation.

### *De novo* Transcriptome Assembly

The FastQC software was used to assess the quality of the raw reads^1^. Then, Illumina adapters, indexes and low-quality reads were removed with Trimmomatic version 0.36 (Andrews et al., 2013; Bolger et al., 2014). Clean reads with a Phred33 score > 30 and length > 36 bp were used in subsequent analysis. The testis reference transcriptome was assembled *de novo* (including all the libraries) using Trinity version 2.4.0 (Grabherr et al., 2011) with default settings except for the no_bowtie option. The raw reads from each library are available in the Sequence Read Archive database (SRA) with Accession No. SRR7880397 to SRR7880408 and the assembled contigs are available in TSA database with Accession No. GGXQ00000000 in BioProject: PRJNA492175 at the National Center for Biotechnology Information (NCBI, United States^2^).

### Functional Annotation

Homology searches were carried out against UniProt release 2018_02 database, and Non-redundant protein (Nr) databases release 2017_09 using Blastx (version NCBI-blast-2.7.1+) software with a cut-off E-value of 1e-05 (Camacho et al., 2009). Gene ontologies were further analyzed using Blast2GO software (version 4.1.9) (Conesa and Götz, 2008) with default parameters to identify the best-represented biological processes detected in the reference transcriptome, based on the number of sequences included in each gene ontology (GO) category. The Kyoto encyclopedia of genes and genomes (KEGG) database was used to identify the transcripts involved in different metabolic pathways (Kanehisa and Goto, 2000).

### Differential Expression Analysis

The reads from each library were aligned back to the reference transcriptome with Bowtie2 version 2.3.4.1 (Langmead and Salzberg, 2012). The estimation of transcripts abundance and normalization [fragments per kilobase million (FPKM)] was carried out with RSEM version 1.3.0 (Li and Dewey, 2011). The matrix built with the FPKM of all libraries was analyzed to obtain the differential expressed transcripts (DETs) among treatments with DeSeq2 (False discovery rate, FDR < 0.05, fold change > 2) (Love et al., 2014). TransDecoder v5.5.0 (Haas et al., 2013) was used to predict the longest open reading frame (ORF) for each differentially expressed transcript, using default parameters. Four experimental conditions were defined to understand the relationship between the reproductive condition and the thermal stress. Each treatment was compared (24POST, 30PRE, and 30POST) against the control treatment (24PRE). The DETs were arranged in clusters according to their expression pattern and represented in a heatmap in R software. The complete differential expression analysis was performed using the Perl and R scripts included in the Trinity package^3^. Shared and exclusive transcripts among treatments were analyzed via Venn diagrams using VennDiagram package in R software.

### Gene Ontology (GO) Enrichment Analysis

The GO enrichment analysis for each transcript was performed to identify the possible biological processes in which these transcripts participate. An enrichment analysis (Fisher’s exact test) was realized in Blast2GO to identify the best-represented categories in the biological process terms (*p*-value < 0.001).

### Quantitative Relative Expression by Real-Time PCR

Thirteen DETs were selected for real-time PCR analysis in a CFX-96 system (Bio-Rad, United States) to validate the transcriptomics results. These transcripts were selected according to two criterions: (a) their significant high expression and (b) their importance in processes involved in the stress response and the reproductive processes.

We used the same RNA samples that were used for sequencing, and additionally, the samples obtained from males exposed to intermediate temperature (28°C), PRE and POST mating conditions were included. cDNA was synthesized using ImProm-II^TM^ Reverse Transcription System (PROMEGA) with 1.0 μg of total purified RNA in a total reaction volume of 20 μL (50 ng/μl) following the manufacturer’s protocol. The obtained cDNA′s were stored at -20°C until use for PCR reactions. Gene-specific primers were designed using Primer3web software v.4.1.0 (Koressaar and Remm, 2007; Untergasser et al., 2012) based on RNA-Seq transcripts sequences. The primers sequences for each selected transcript are shown in Supplementary Table 1. The efficiency of the target and reference transcripts were calculated from a standard curve with an initial dilution factor of 1:5 and six subsequent serial dilutions with a factor of 1:2 of a cDNA pool including all experimental conditions. In our study, genes commonly used in the literature as housekeepings in other organisms (for example tubulins, elongation factors, actins, Glycerol-3-phosphate dehydrogenase) were significantly differentially expressed among the experimental conditions. For this reason, the housekeeping transcripts used in this study were chosen from the annotated transcript database of the assembled reference transcriptome. The selection was based on the lack of differential expression among treatments (FDR value = 1).

A total of 10 potential reference transcripts were evaluated for expression stability with the Genorm, Normfinder and Bestkeeper software, which results indicated that TUFM and TUBGCP were the most stable transcripts and were used as housekeeping transcripts for the relative expression analysis.

The qPCR reactions were carried out by triplicate with homemade Evagreen Mix 2x (Evagreen 20,000x in water, Biotium and AccuStart Taq DNA polymerase, Quanta, Beverly, MA, United States). The reaction consisted in 5 μL of Evagreen Mix 2x, 0.2 μM of forward and reverse primers, 3 μL of cDNA template (dilution 1:5 or equivalent to 30 ng of total RNA) and 1.6 μL of sterile, free nuclease water, for a final volume of 10 μL. The thermal cycling conditions were 94°C for 3 min, followed by 40 cycles at 94°C 30 s, annealing temperature for 30 s (Supplementary Table 1) and 72°C for 30 s. A melt curve analysis was included at the end (95°C for 10 s, 65°C to 95°C for 5 s, with increments of 0.5°C) to corroborate PCR products specificity. The amplicons length were confirmed in agarose gel electrophoresis at 1.5%. Relative expression (RE) of target transcripts was estimated using the ΔΔ Cq method, as proposed by Hellemans et al. (2007). For statistical analysis, all RE values were transformed to logarithm (log10), and a two-way ANOVA model was used to establish the effects of temperature and mating condition, with a statistical significance of *P* < 0.05. A *post hoc* analysis of means was done using Fisher’s LSD test. All statistical analyses were performed using STATISTICA 6.1 (StatSoft, Tulsa, OK, United States).

## Results

### Transcriptome Sequencing, Trimming, and Assembly

The sequencing of all the testis libraries generated 53,214,611 paired-end raw reads with a length of 75 bp. After discarding Illumina adaptors and reads with low quality, a total of 48,101,426 reads with a Phred score over 30, were *de novo* assembled to generate the reference transcriptome using Trinity. Table 1 summarizes the number of sequenced reads and the trimming statistics per sample.

**Table 1:** RNA-Seq reads obtained on Illumina MiSeq system.

Sequencing statistics	Number of raw reads before trimming	Number of raw reads after trimming	Raw reads after trimming (%)
24PRE-1	4,261,482	3,715,125	87.18
24PRE-2	3,801,367	3,514,073	92.44
24PRE-3	3,783,317	3,509,953	92.77
24POST-1	4,144,156	3,634,299	87.70
24POST-2	4,529,867	3,997,421	88.25
24POST-3	3,908,012	3,596,089	92.02
30PRE-1	5,792,353	5,164,041	89.15
30PRE-2	4,160,642	3,705,038	89.05
30PRE-3	4,933,884	4,580,123	92.83
30POST-1	4,648,617	4,135,854	88.97
30POST-2	4,822,915	4,452,090	92.31
30POST-3	4,427,999	4,097,320	92.53
Total	53,214,611	48,101,426	90.39


The *de novo* assembled testis transcriptome consisted in 53,848,027 bases. The contigs length ranged from 201 nt to 12,758 nt with an average length of 631 nt, N50 = 889 nt (based on all transcript contigs) and GC content of 38%. A total of 85,249 transcripts (including all isoforms) and 77,661 genes were reconstructed (Table 2). The *de novo* testis transcriptome of *O. maya* was deposited at the NCBI.

**Table 2:** *De novo* assembly and annotation statistics.

Trinity assembly statistics	All contig transcripts
Total assembled bases	53,848,027
Total number of transcripts	85,249
Total number of genes	77,661
GC content (%)	38
Contig N10	2,949
Contig N20	2,118
Contig N30	1,604
Contig N40	1,204
Contig N50 (based on all transcript contigs)	889
Contig N50 (based on longest contig isoform)	783
Median contig length (nt)	381
Average transcript length (nt)	631
Total transcripts with ORF	14,331
Maximum length (bp)	12,758
Minimum length (bp)	201
Number of transcripts over 1 kb	14,331
Annotation statistics	
Annotated transcripts by UniProt	13,154 (15.4%)
Contigs with cellular component terms	10,856 (82.5%)
Contigs with biological process terms	10,663 (81.1%)
Contigs with molecular function terms	10,535 (80.1%)
Annotated transcripts by Nr	11,151 (13.1%)
Annotated transcripts by KEGG	5,461 (6.4%)


**FIGURE 1:**
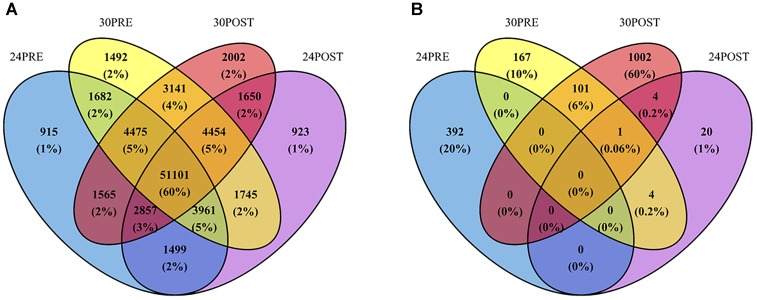
**(A)** Venn diagram of the number of transcripts expressed in the reference testis transcriptome of *Octopus maya* in each treatment. **(B)** Venn diagram of DETs with significant higher expression in each treatment. Treatments: 24PRE – control treatment exposed to 24°C; 24POST – mated and exposed to 24°C; 30PRE – exposed to 30°C; 30POST – mated and exposed to 30°C.

From the reference testis transcriptome, a total of 915 transcripts were exclusively expressed in the control treatment (24PRE), 923 in 24POST, 1,492 in 30PRE and 2,002 in 30POST (Figure 1A).

### Functional Annotation of *O. maya* Testis Transcriptome

**FIGURE 2:**
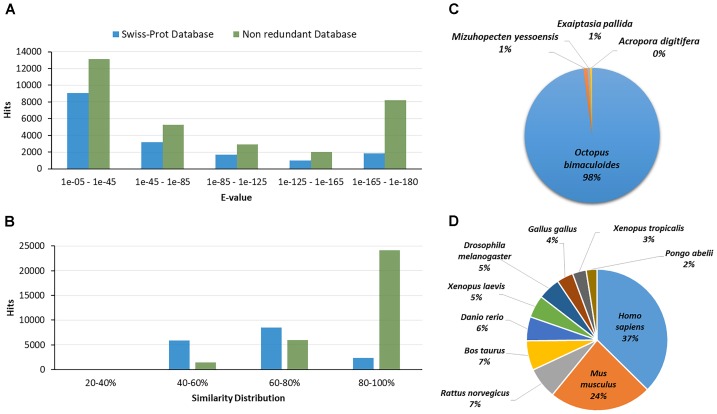
**(A)** E-value distribution of the Blastx hits against the UniProt and Non-redundant (Nr) database for each transcript. **(B)** Similarity distribution of the Blastx hits against the UniProt and Nr database. **(C)** Species distribution of the top blast hits of the transcripts in the Nr database in the testis transcriptomic analysis of *O. maya* males. **(D)** Species distribution of the top blast hits of the transcripts in the UniProt database in the testis transcriptomic analysis of *O. maya* males.

The transcripts were annotated by comparing with Nr, UniProt and KEGG databases. In total, 16,804 (19.7%) and 31,555 (37.0%) transcripts had at least one significant homolog against proteins of the UniProt and Nr databases, respectively (e-value cut-off: 1e-5). Most of the sequences with homology against those databases had an e-value among 1e-05 to 1e-45. The 51% of the homologous sequences obtained from UniProt presented a similarity distribution among 60 to 80%, while the 76% of the homologous found in the Nr had a similarity distribution among 80–100%. Figures 2A,B show the e-value and the similarity distribution for the blastx hits against UniProt and Nr databases. From the blast hits obtained with the UniProt database, the higher number of matches corresponded with sequences of *Homo sapiens* (37%) followed by sequences of *Mus musculus* (24%), *Rattus norvegicus* (7%), and *Bos taurus* (7%) (Figure 2D). In the case of the hits matched with the Nr database, the highest number of matches corresponded to sequences of *Octopus bimaculoides* (96%) (Figure 2C). This high identity percentage suggests that the *O. maya* gene fragments were correctly assembled and annotated.

We applied the Blast2GO algorithm to classify the transcripts in functional categories: biological process, molecular function, and cellular component. The results showed that only 13,154 transcripts (15.4%, UniProt database) and 11,151 (13.1%, Nr database) of 85,249 transcripts had at least one GO term assignation and could be annotated (Table 2).

The GO assignments carried out at level three revealed that most of the sequences were categorized in cellular components (10,856; 82.5%), followed by biological processes (10,663; 81.1%) and molecular functions (10,535; 80.1%). The biological processes identified were cellular metabolic process (6,764 transcripts; GO:0044237), response to stress (1,184 transcripts; GO:0006950), cell cycle (1,279 transcripts; GO:0007049), microtubule-based process (530 transcripts; GO:0007017), response to abiotic stimulus (445 transcripts; GO:0009628), cell motility (444 transcripts; GO:0048870), chromosome segregation (174 transcripts; GO:0007059), immune response (316 transcripts; GO:0006955), sperm part (64 transcripts; GO:0097223), and meiotic cell cycle (90 transcripts; GO:0051321). The main cellular components identified were intracellular (9,594 transcripts; GO:0005622), membrane-bounded organelle (6,855 transcripts; GO:0043227), endomembrane system (2,026 transcripts; GO:0012505), and protein complex (1,640 transcripts; GO:0043234). The main molecular functions identified were protein binding (5,139 transcripts; GO:0005515), hydrolase and transferase activity (2,448 and 2,257 transcripts, respectively; GO:0016787 and GO:0016740; Figure 3).

**FIGURE 3:**
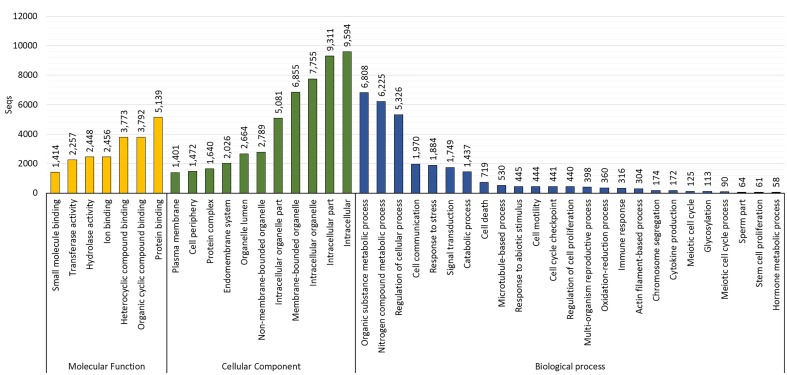
Gene ontology (GO) distribution by category at level 3 in the reference testis transcriptome of *O. maya* males.

A total of 3,317 transcripts (25.2%) matched with homologous proteins in the KEGG database associated with 130 distinct KEGG pathways. Among the top five categories, nucleotide, cofactors, and vitamins metabolism are the largest represented classes, and the top-hits pathways in these categories were purine and thiamine metabolism with 705 transcripts.

### Differentially Expressed Transcripts (DETs)

**FIGURE 4:**
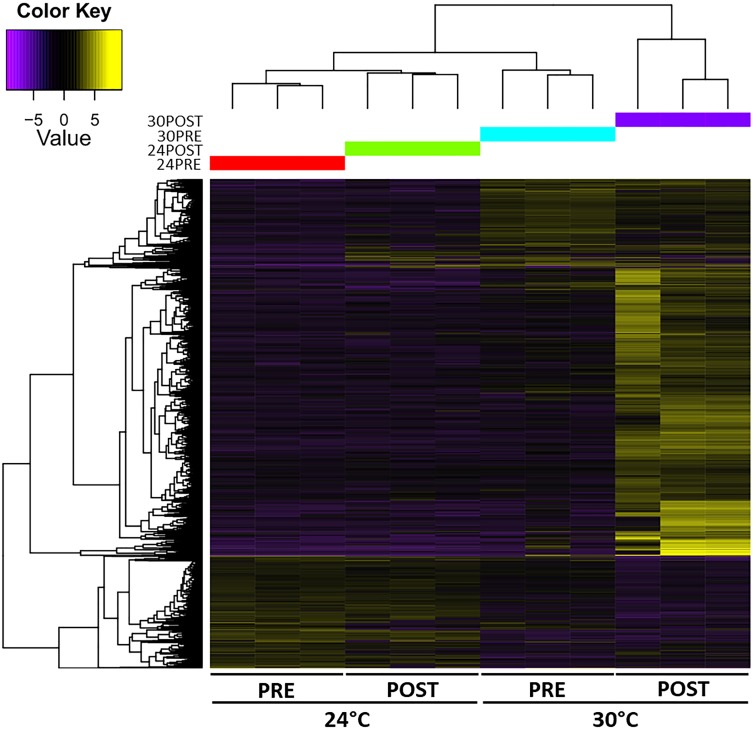
Heatmap of the abundance of differentially expressed transcripts (rows, FDR < 0.05, Fold change > 2) in the *O. maya* testis transcriptome in each treatment (columns). The dendrogram shows that temperature modulated the expression patterns. Treatments: 24PRE – control treatment exposed to 24°C; 24POST – mated and exposed to 24°C; 30PRE – exposed to 30°C; 30POST – mated and exposed to 30°C.

The heatmap of DETs detected in each library is shown in Figure 4. The expression patterns revealed that 24PRE and 24POST had a similar expression pattern, which evidences that copula did not affect gene expression under optimal thermal condition. In 30PRE treatment, it was observed some transcripts (273) with significantly higher expression in comparison to the control treatment (24PRE). At this point, we found that thermal increment modifies the expression patterns in the testis. In the case of 30POST, we observed the major number of transcripts with higher expression in comparison to the control treatment. The 30POST condition showed an expression profile entirely different for the control, where a significant number of transcripts were upregulated meanwhile under normal conditions these same transcripts are downregulated. This pattern evidences that the combined effect of high temperatures and the reproductive activity has a significant effect over the molecular mechanisms that regulate gene expression in the testis of *O. maya* males.

The differential expression analysis showed 1,881 significantly differentially expressed transcripts using 24PRE as the control treatment. A total of 1,410 transcripts (1,114 coding transcripts and 296 non-coding, Supplementary Table 4) were significantly more abundant (29 transcripts in 24POST, 167 transcripts in 30PRE and 1,002 transcripts in 30POST; *P* < 0.05, FC > 2) in all treatments vs. the control. Figure 1B shows the Venn diagram of the transcripts with significantly higher expression in the treatments that had homologs with the UniProt database. A total of 471 (378 coding transcripts and 93 non-coding, Supplementary Table 4) transcripts were significantly more abundant in the control vs. all the treatments (16 transcripts vs. 24POST, 160 transcripts vs. 30PRE and 295 transcripts vs. 30POST; FDR < 0.05, FC > 2). We also found differentially expressed transcripts that did not match the protein databases, a total of 13 in 24POST, 176 in 30PRE and 977 in 30POST. Even, some of these transcripts have higher expression than those putative transcripts with homologs in the peptide databases. The longest ORF region of the DETs validated in qPCR are shown in Table 3.

**Table 3:** Longest ORF regions of the differential expressed transcripts used in qPCR.

Contig ID	Transcript encoding	Start	End
TRINITY_DN7707_c0_g1_i1	CASP7	359	1126
TRINITY_DN16555_c1_g1_i2	CHD5	3	4217
TRINITY_DN3007_c0_g1_i1	DNAJB13	101	1051
TRINITY_DN26602_c0_g1_i1	GPX4	280	1437
TRINITY_DN33756_c0_g1_i1	HSPA9	3	2150
TRINITY_DN2386_c0_g1_i1	HTT	1	3105
TRINITY_DN16585_c0_g1_i1	KLHL10	479	2116
TRINITY_DN13245_c0_g1_i1	MIF	65	409
TRINITY_DN11092_c0_g1_i1	NFKB2	98	2449
TRINITY_DN7210_c0_g1_i1	RABL2A	342	881
TRINITY_DN9963_c0_g1_i1	TDRD1	311	2029
TRINITY_DN17275_c0_g1_i1	TSSK2	849	1205
TRINITY_DN17130_c0_g1_i2	ZMYND15	297	1823
TRINITY_DN35138_c0_g1_i1	TUFM	3	1382
TRINITY_DN14290_c0_g1_i1	TUBGCP	312	2627


### GO Enrichment Analysis

**FIGURE 5:**
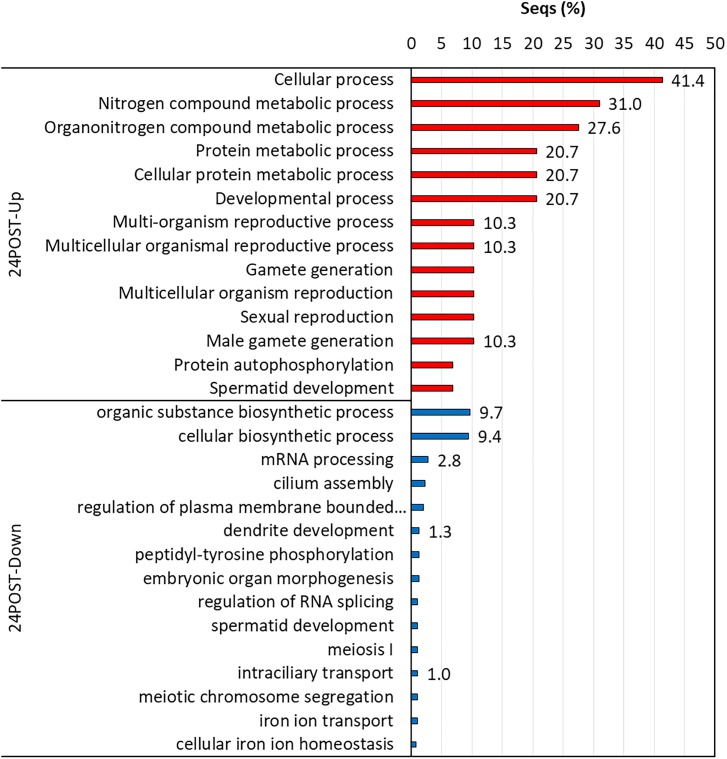
Enriched GO terms of biological processes (Fisher exact test, FDR < 0.05) in 24POST treatment vs. the control treatment (24PRE). Up (red) and down-regulated (blue) transcripts are shown.

**FIGURE 6:**
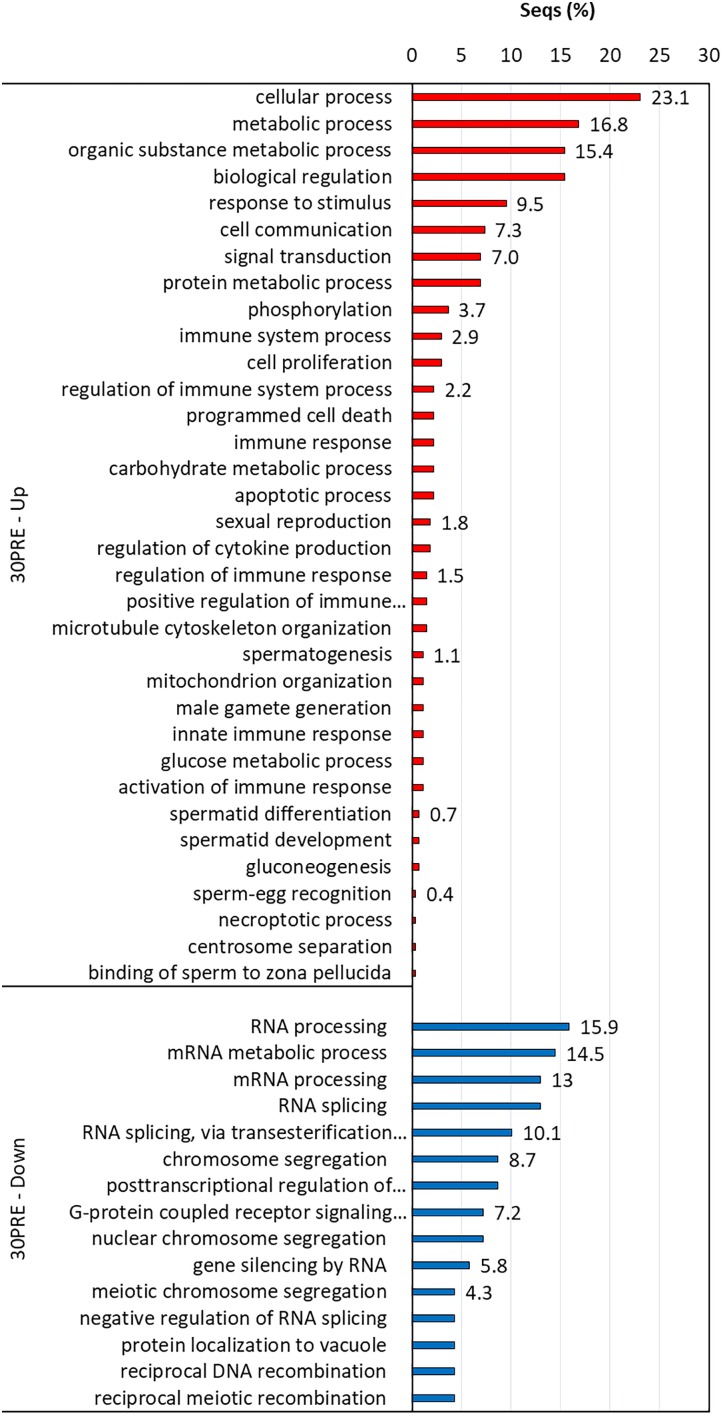
Enriched GO terms of biological processes (Fisher exact test, FDR < 0.05) in 30PRE treatment vs. the control treatment (24PRE). Up (red) and down-regulated (blue) transcripts are shown.

**FIGURE 7:**
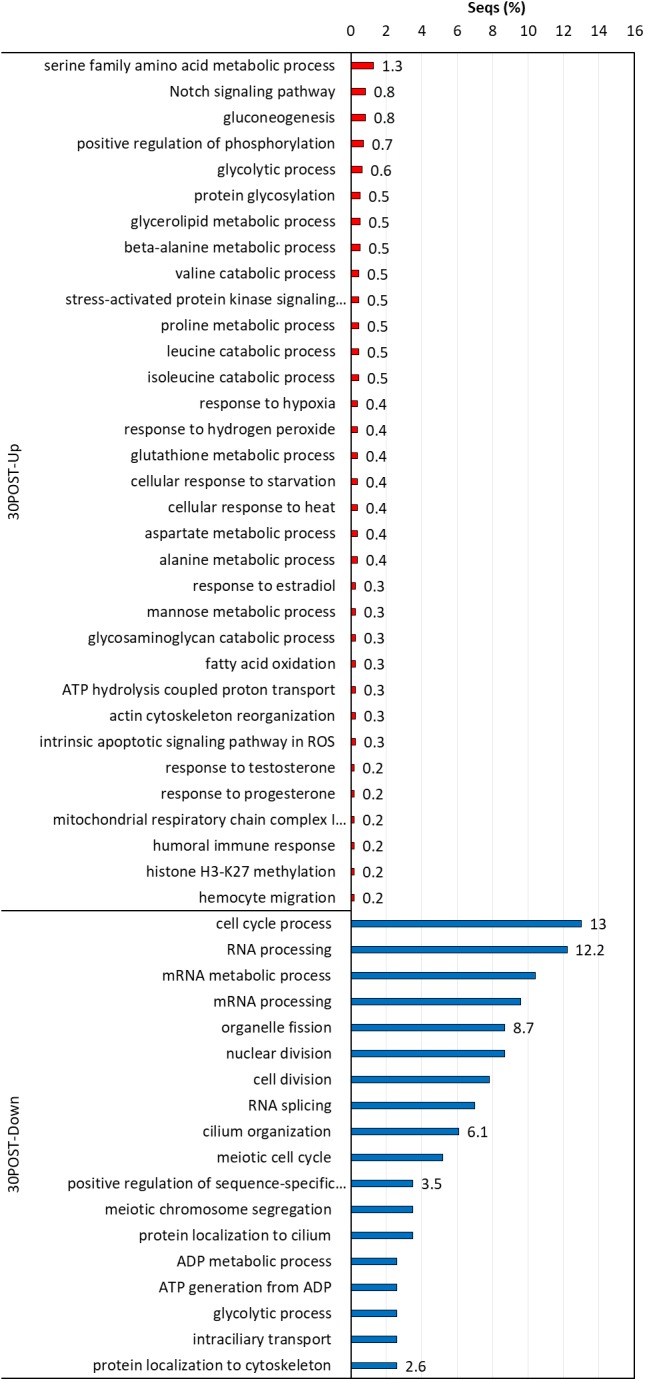
Enriched GO terms of biological processes (Fisher exact test, FDR < 0.05) in 30POST treatment vs. the control treatment (24PRE). Up (red) and down-regulated (blue) transcripts are shown.

Biological processes with significant enrichment (*P* < 0.05) were detected in each thermal and reproductive condition by using the transcripts with higher expression in each treatment. In 24POST, 16 of the 29 upregulated DETs significantly enriched 146 biological process categories (Figure 5), while nine of the 16 downregulated DETs significantly enriched a unique biological process category. In 30PRE, 96 of the 273 upregulated DETs significantly enriched 396 biological processes (Figure 6), while 76 of the 160 downregulated DETs significantly enriched21 biological processes. In 30POST, 531 of the 1,108 upregulated DETs enriched 390 biological processes significantly (Figure 7), while 129 of the 295 downregulated DETs significantly enriched 67 biological process categories. The transcripts that best represented the enriched biological processes involved in stress response and reproductive process are shown in Supplementary Tables 2, 3.

### Stress Response

#### Transcripts Involved in Response to Thermal (TS) and Oxidative Stress (OS)

**FIGURE 8:**
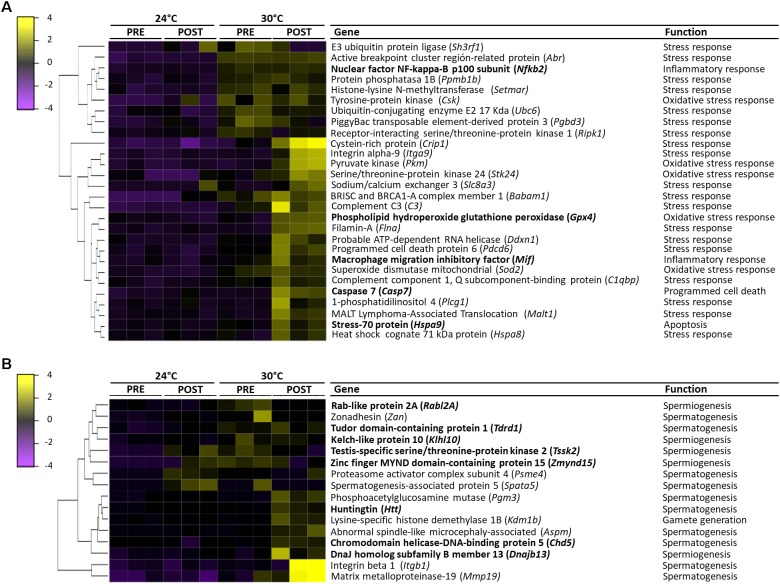
Heatmap representing the expression values of differentially expressed transcripts (FDR < 0.05, Fold change > 2) between 24PRE, 24POST, 30PRE, and 30POST treatments and their main function. **(A)** Transcripts involved in the stress response. **(B)** Transcripts involved in reproductive process. Sample names are represented in columns and significant transcripts are represented in rows. Transcripts are clustered together based on expression similarity. Low to high expression is represented by a change of color from purple to yellow, respectively.

All the differentially expressed transcripts involved in the thermal stress response were highly expressed at 30°C (Figure 8A). In the 30PRE condition, the transcript encoding SH3RF1 gene (TS) showed higher expression in comparison to 30POST. The transcripts encoding genes such as CRIP1, ITGA9, SLC8A3, FLNA, DDXN1, and PDCD6 (ST) were conspicuous in 30POST condition. The transcripts encoding ABR, SETMAR, UBC6, BABAM1, and C3 genes (TS) were induced in both conditions.

The transcripts involved in the oxidative stress response as CSK and SOD2 were highly abundant in both treatments (30PRE and 30POST), while the transcripts encoding PKM, STK24, GPX4, and SOD2 genes were highly expressed specifically in 30POST treatment (Figure 8A).

#### Transcripts Involved in Cytokine Production, Inflammatory Process, Apoptosis, and Necroptosis

The transcripts that best-represented these categories were exclusively induced at 30°C and specific changes were observed between PRE and POST condition (Figure 8A). The transcripts involved in the cytokine production as PPMB1, CSK, and PGBD3 showed higher expression in both 30PRE and 30POST conditions, meanwhile C3, and BCL only in 30POST condition. The transcripts up-regulated in inflammatory processes as MIF, MAPKAPK2, and CHIA presented higher expression in 30POST and NFKB2 only in 30PRE condition. The transcripts encoding HSPA9, TRAF2 and TNIP2, showed higher expression in 30POST condition, and SH3GLB1 and TFDP1 in 30PRE. The transcript encoding RIPK1 (necroptotic process) presented higher expression in 30PRE condition. The transcripts encoding CASP7 gene was highly abundant in the condition 30POST.

### Reproductive Processes

#### Transcripts Involved in Spermatogenesis, Spermiogenesis, and Gamete Generation Processes

The transcripts that best-represented these biological processes were exclusively induced at 30°C and specific changes were observed between PRE and POST condition (Figure 8B). The transcripts involved in the spermatogenesis process as ZAN and TDRD1 showed higher expression at 30°C under PRE and POST conditions meanwhile PSME4 showed higher expression in 24POST and 30PRE treatments. SPATA5 showed unique higher expression in 24°C after copula. The up-regulated transcripts PGM3, HTT, ASPM, CHD5, ITGB1 and MMP19, showed the highest expression in 30POST treatment. The transcripts involved in spermiogenesis as RABL2, KLHL10, and TSSK2 showed higher expression in 30°C PRE and POST conditions meanwhile ZMYND15 showed higher expression in 24POST and 30PRE treatments. The up-regulated DNAJB13 transcript showed the highest expression in 30POST. The KDM1B transcript (gamete generation process) was highly expressed in 30POST treatment.

#### qPCR Validation

The Pearson correlation coefficient was measured in the RNA-Seq and qPCR data. The correlation coefficient for GPX (*r* = 0.91), HSPA9 (*r* = 0.90), CASP7 (*r* = 0.99), NFKB2 (*r* = 0.95), and MIF (*r* = 0.94) revealed that relative expression measured by qPCR is consistent with the RNA-Seq data (Supplementary Figure 1). The expression patterns of the up-regulated transcripts involved in the reproductive process were confirmed by qRT-PCR (Supplementary Figure 2). The results revealed high correlation values for KLHL10 (*r* = 0.99), HTT (*r* = 0.99), TDRD1 (*r* = 0.80), and RABL2A (*r* = 0.83).

**FIGURE 9:**
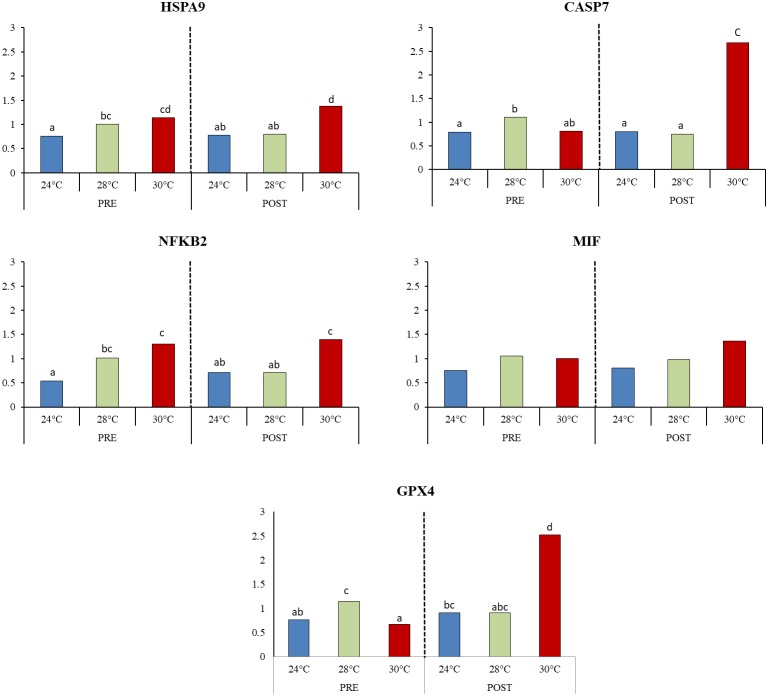
Relative expression of transcripts associated with thermal stress response. Mean values (back-transformed from logarithms) are shown in bars and letters are used to denote differences between means (*P* < 0.05).

Different transcripts presented in oxidative stress response (GPX4), inflammatory processes (MIF, NFKB2) apoptosis (CASP7, HSPA9) were selected for relative expression analysis by qPCR to validate the differential expression results described above. Consistent with the RNA-Seq data, transcripts encoding the genes GPX4, CASP7, HSPA9, and MIF showed a higher expression in 30POST condition. Meanwhile, transcript NFKB2 presented the highest expression at 30°C compared to 24°C PRE condition. Additionally, when the intermediate temperature was included in the expression analysis, ANOVA results for stress response-related transcripts (Figure 9) indicated that temperature has a significant effect on the expression levels of GPX4 (*P* = 0.0029), CASP7 (*P* = 0.0005), HSPA9 (*P* = 0.0026), and NFKB2 (*P* = 0.0006). On the other hand, differences between mating condition were detected only for GPX4 (*P* = 0.0003) and CASP7 (*P* = 0.0085), with the interaction between temperature and condition being also significant for both transcripts (*P* < 0.05). This result was mainly caused by the expression in PRE 28°C, which was significantly higher than that at 24 and 30°C in the same reproductive condition. Meanwhile, in POST condition, the expression was significantly higher at 30°C respectively to that observed at 24 and 28°C. No significant differences between mating conditions were observed for HSPA9 (*P* = 0.96) and NFKB2 (*P* = 0.99), nor for the interaction between factors (*P* > 0.05). Finally, the relative expression of MIF did not show significant differences between temperature (*P* = 0.067), condition (*P* = 0.44) or the interaction between them (0.537), although its expression appeared to be increased with temperature in both conditions.

**FIGURE 10:**
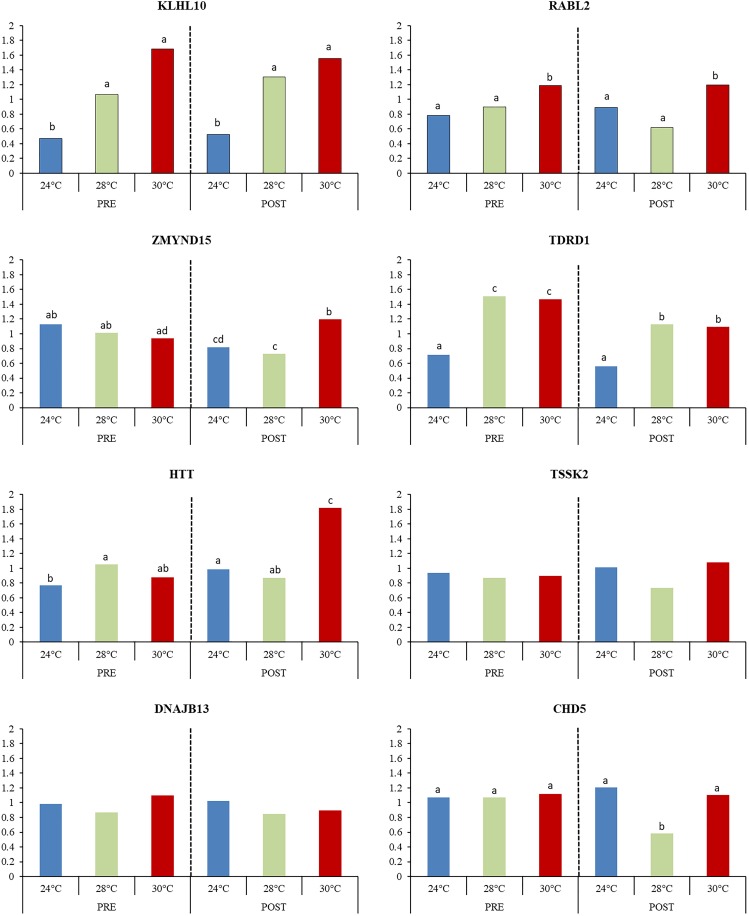
Relative expression of transcripts associated with male reproductive process. Mean values (back-transformed from logarithms) are shown in bars and letters are used to denote differences between means (*P* < 0.05).

The expression pattern of the eight highly expressed transcripts involved in the reproductive process (KLHL10, TSSK2, DNAJB13, RABL2A, CHD5, ZMYND15, TDRD1, and HTT) were confirmed by qRT-PCR (Figure 10). For HTT, the highest expression was observed in 30POST, whereas for TDRD1 and KLHL10 both PRE and POST condition showed the highest expression. In the case of RABL2A, 30PRE condition showed higher expression than 24PRE. All these results were consistent with differential expression analysis of the transcriptome.

In order to determine the role of temperature and mating condition in *O. maya* in the expression pattern of the selected transcripts, the 28°C temperature was included in the analysis. This temperature allowed to find that there was a significant effect of temperature in the expression of KLHL10 (*P* < 0.01), RABL2A (*P* = 0.009), CHD5 (*P* = 0.025), TDRD1 (*P* < 0.01), ZMYND15 (*P* = 0.036), and HTT (*P* = 0.002). However, different patterns were observed regarding the reproductive condition and the interaction between the factors. For KLHL10 and RABL2, no effect of mating condition (*P* > 0.05) or the interaction between factors (*P* > 0.05) was observed, indicating that expression between temperatures has similar patterns in PRE and POST mating, being significantly higher in average at 30°C for both transcripts. On the other hand, for CHD5 no significant differences between PRE and POST condition were observed (*P* = 0.136), but the significance of the interaction between temperature and mating conditions (*P* = 0.0352) was caused by the significantly lower expression at 28°C in POST condition. For TDRD1 the expression in PRE condition was significantly higher on average respect to POST (*P* = 0.001), but no significant interaction was observed (*P* = 0.944) indicating that for PRE and POST condition the expression of TDRD1 has a similar pattern between temperatures with the lowest expression observed at 24°C.

## Discussion

*Octopus maya* as an ectotherm organism is strongly influenced by temperature (Regil et al., 2015). Temperature plays an important role in different life aspects as embryo development, growth patterns, morphology, physiology, and reproduction. As an endemic species of the YP, is influenced by the thermal characteristics of the platform, where temperatures can vary since 21 to 30°C along the year (Noyola et al., 2013a,b). In this study, male octopuses were exposed at three temperatures 24°C (Optimal), 28°C (intermediate) and 30°C (Stress) during 30 days, and then, a group of males for each experimental temperature were mated with females acclimated at 24°C. An RNA-Seq analysis of the testis transcriptome at contrasting temperatures (24 and 30°C) was realized to evaluate the transcriptomic responses to chronic thermal stress and the mechanisms involved in the regulation of reproductive processes. In recent years, the high-throughput sequencing techniques have allowed obtaining genetic and genomic information of both model and non-model organisms, the latter in which there are no (or very limited) genomic resources (Ekblom and Galindo, 2011). This technique allows evaluating the expression profiles of a large number of genes, robustly. In our RNA-Seq analysis, we used pooled samples to minimize the effects of biological variation in treatments. We obtained the *de novo* transcriptome of *O. maya* testis constituted by 85,249 transcripts reconstructed. The main species that matched our blast hits against UniProt and Nr Databases were *Homo sapiens* and *O. bimaculoides*, respectively.

This match is completely attributable to the big number of known proteins of model organisms like *Homo sapiens* in the UniProt database and in the case of *O. bimaculoides*, the recent release of its genome (Albertin et al., 2015), and the close phylogenetic relationship between *O. maya* and *O. bimaculoides* (Juárez et al., 2012). The GO functional annotation showed 13,154 (15.4%) and 11,151 (13.1%) transcripts with homologies in the UniProt and Nr databases, with similar proportion to that found in other cephalopods as *Octopus vulgaris* (Zhang et al., 2012; Castellanos-Martínez et al., 2014), *Euprymna tasmanica* (Salazar et al., 2015), and *Sepia officinalis* (Cornet et al., 2014). The fact that the 84.6 and 86.9% (UniProt and Nr databases) of the transcripts did not match any known proteins suggests that there may be a high number of potentially uncharacterized transcripts in *O. maya* that remain to be properly characterized. In this study, 1,166 transcripts without homology were differentially expressed among treatments with high expression values; more studies have to be done to characterize these uncharacterized transcripts. This lack of molecular data emphasizes the importance of cephalopods studies to elucidate the molecular mechanisms involved in their physiology, development, growth, and reproduction.

In general, in the reference testis transcriptome, we identified putative transcripts involved in the biological process as metabolism, stress response, cell cycle, microtubule-based process, sperm part, and chromosome segregation. These results indicated that some important traits inherent to the organisms as metabolic activity, cellular response, and cellular processes occurred in *O. maya* testis during chronic thermal stress and mating activity.

### Transcripts Related to Thermal Stress Response in *O. maya* Testis Transcriptome

One of the goals in the present study was to assess the presence of transcripts involved in stress response, apoptosis, and inflammatory processes in the *O. maya* testis transcriptome to confirm that thermal stress affects the molecular mechanisms that regulate the reproductive performance and success of this species. There are different heat stress response mechanisms as DNA reparation, heat shock response, antioxidant defense, cell cycle checkpoints, and apoptosis (Pérez-Crespo et al., 2008). The apoptosis process in the testis is characterized by the apparition of acidophilic bodies. These acidophilic bodies under normal conditions of spermatogenesis, maintain the equilibrium between cellular proliferation and apoptotic degeneration (Lin et al., 1997). Apoptosis process has been well-understood in humans with infertility issues, where an increment in the process such as maturity arrest and hypospermatogenesis has been observed (William et al., 1997). In this study, we identified necroptotic processes in male octopus exposed to 30°C; this is coincident with the severe testicular damage observed at 30°C, an increment four times higher than that of those exposed to 24°C, and dilation of germ cells strata (López-Galindo et al., 2019). The necrosis is a process of programmed cell death caused by external factors which trigger an immune response characterized by the inflammatory process (Shaha et al., 2010). In this sense, the gene ontology analysis of the *O. maya* testis transcriptome revealed the presence of transcripts involved in regulation of cytokine production (PPMB1, C3, CSK, PGBD3, BCL3) which are cell signaling proteins that regulate the inflammation and infection in the body (Castellanos-Martínez et al., 2014); inflammatory process which is important for a rapid and efficient elimination of damaged tissue (NFKB2, MIF, MAPKAPK2, CHIA2) (Ottaviani et al., 2010); apoptosis which is a process characterized by dying cells, cytoplasmatic shrinkage, active membrane blebbing, chromatin condensation, and typically, fragmentation into membrane-enclosed vesicles or apoptotic bodies (HSPA9, SH3GLB1, TFDP1, TRAF2, TNIP2); and necroptotic process where necrosis is characterized by cytoplasmic and organelle swelling, and plasma membrane rupture (RIPK1) (Peterson et al., 2015). The necroptosis has been recently investigated. This form of necrosis is dependent of the kinases RIP1 and RIP3, and a pseudokinase MLKL (Peterson et al., 2015). The differential expression of these transcripts can explain the presence of fourfold acidophilic bodies at 30°C, compared to the other treatments, in addition to basophilic material, and vacuolated basal compartments (López-Galindo et al., 2019).

The heat shock proteins (HSP’s) are a group of functionally related proteins present in all living organisms. Among other important roles, the HSP’s are involved in protein folding and unfolding, and their expression is induced by increasing temperature as well as other stresses (Wang et al., 2014). The upregulation of HSP genes constitutes the core part of the cellular heat shock response (Wang et al., 2014). We identified six members of three HSP families: HSP20 (HSPB6), HSP40 (DNAJB13), and HSP70 family (HSPA9, HSPA12A, HSP70B2, and HSPA8) involved in heat stress response. HSP’s as the stress-70 protein, mitochondrial (HSPA9) were highly expressed on conditions of high temperature. The family of the HSP 70 is one of the most highly conserved of the HSP’s. They function as molecular chaperones that act as a first defense line and mediate the refold of stress-denatured proteins, prevent the aggregation of denatured proteins and limit the cellular damage (Guzman and Conaco, 2016). HSP’s protect the cell from de deleterious effects of heat and module the stress response (Castellanos-Martínez et al., 2014). The analysis of relative expression by qPCR revealed that HSPA9 transcript had a high-level expression in organisms exposed to 28 and 30°C in the PRE condition, while in the POST condition this transcript had a high relative expression at 30°C in comparison to 24 and 28°C. Since this protein plays a role in cell proliferation, stress response and maintenance of the mitochondria, HSPA9 could be playing an important role in the preservation of mitochondria during thermal stress, which is of vital importance.

The caspase-7 (CASP7) is an executioner caspase that degrades cellular components. The caspase proteins constituted the core of apoptotic machinery (Castellanos-Martínez et al., 2014). Caspases have been described in vertebrates. However, there is limited information in invertebrates such as the abalone *Haliotis diversicolor* and the mussel *M. galloprovincialis*, but there are just a few studies in cephalopods such as the common octopus *O. vulgaris* (Romero et al., 2011; Castellanos-Martínez et al., 2014). In our study, CASP7 was induced under both thermal stress and mating, presenting its higher relative expression in 30POST treatment. This expression pattern could indicate that apoptotic mechanisms have been executed in the testis of *O. maya* males under chronic thermal stress and corroborate the findings of severe tisular damage at high temperatures observed by López-Galindo et al. (2019).

The nuclear factor NF-kappa-B p100 subunit (NFKB2) is an inducible transcription factor that plays a central role in the inflammatory response and immune function, which is activated quickly by a wide group of agents and cell stress (Srikanth et al., 2017; Sun, 2017). It seems that NF-Kb is an innate immune system pathway evolutionarily conserved and present in mollusks (Castellanos-Martínez et al., 2014). NFKB2 presented significant higher relative expression under chronic thermal stress at 28 and 30°C (Srikanth et al., 2017; Sun, 2017). This response confirms the inflammatory processes observed in the testis in both temperatures by López-Galindo et al. (2019). In Holstein’s calves, NFKB2 has been identified as an important transcription factor that modulates the heat stress response (Srikanth et al., 2017).

The macrophage migration inhibitory factor (MIF) is a multifunctional protein which acts as a pro-inflammatory cytokine, a pituitary hormone, immunoregulator, and mitogen (Anahara et al., 2008). MIF transcript did not show significant differences in relative expression levels between treatments, which could be related to the multifunctional role of this protein (Anahara et al., 2008). However, a positive relationship to temperature was observed.

The Phospholipid hydroperoxide glutathione peroxidase (GPX4) protects cells against membrane lipid peroxidation and cell death (Imai et al., 2009). GPX4 transcript showed a significant relative expression in organisms of 28PRE and its highest expression was observed in organisms of 30POST treatment.

The heat stress is a determinant factor that affects the physiology and reproductive performance of the organisms. Pérez-Crespo et al. (2008) mentioned that heat stress affects the sperm viability, sperm motility, reduced the fertilization capacity and survival, temporarily delays embryonic growth and promotes degeneration, causes abnormalities in the chromatin condensation, damage to DNA, RNA, and protein synthesis and denatures proteins. In this study, it was possible to corroborate that thermal stress induces the expression of transcripts involved in the stress response to compensate the damage caused by chronic thermal stress, however, when the effect of mating is added, this expression is increased. Unfortunately, despite these compensatory mechanisms, testicular damage caused by chronic thermal stress at 30°C is severe and directly affects the reproductive success of *O. maya* males. It is important to realize more studies that allow us to elucidate if severe testicular damage could have a gradual return to normal conditions of spermatogenesis.

### Critical DETs Involved in Spermatogenesis and Spermiogenesis Process in *O. maya* Testis Transcriptome

Spermatogenesis is a dynamic and synchronized maturation process from germ cells to mature spermatozoa that take place in the seminiferous tubules in the testis (Shaha et al., 2010). Stringent temporal and spatial expression of genes during both transcriptional and translational processes during protein synthesis is of fundamental importance to ensure the highly ordered processes of spermatogenesis (He et al., 2012). The goal of spermatogenesis is to produce a genetically male gamete that can fertilize an ovum ultimately produce offspring, and this process involves series of intricate, cellular, proliferative, and developmental phases such as mitotic proliferation (proliferation and differentiation of spermatogonia), meiotic phase (differentiation of spermatocytes), and spermiogenesis (differentiation oh haploid germ cells from round spermatids to elongated spermatids and spermatozoa) (Yan et al., 2010; Dang et al., 2012; He et al., 2012). Protein phosphorylation is the most common post-translational protein modification in eukaryotes that controls the spermatogenesis process (Zhang et al., 2010). A protein kinase family, the testis-specific serine/threonine kinases (TSSK’s) may play a role in male spermatogenesis because they are expressed mainly or specifically in the testis. Five members of the TSSK family has been identified in mouse (TSSK1, TSSK2, TSSK3, TSSK3, TSSK4, and TSSK5) (Zhang et al., 2010). Previous research revealed that TSSK2 phosphorylates several flagellar proteins in the central apparatus of the sperm axoneme, such as SPAG16 and testis-specific kinase substrate (Xu et al., 2008; Zhang et al., 2010). The TSSK2 are implied in the formation of microtubule structures during spermatogenesis and is crucial for spermatid production (Zhang et al., 2010). In this study, TSSK2 transcript was identified at 30°C in both PRE and POST conditions. The higher expression was identified in 30PRE condition. Insufficient expression of TSSK2 could interrupt spermiogenesis and results in failure of elongated spermatids, triggering male infertility. This results coincides with the lack of spermatids in the testis of octopus thermally stress and could explain the lack of parental contribution as observed by López-Galindo et al. (2019).

Spermiogenesis involves three subsequent significant events: formation of the acrosome, flagellum formation, and cytoplasm reorganization. Yan et al. (2010) mentioned that meiosis is unique to germ cells, and spermatogenesis is unique to male germ cell development, and this particularity demands unique genes and gene products to execute their functions. The spermatogenesis process implies the use of ∼10% of the entire protein-encoding genes meanwhile spermiogenesis alone involves 500 testis-specific genes (Yan et al., 2010). There are a series of transcription factors that are important to regulate the gene expression. In the present study, we identified the transcriptional repressor ZMYND15 with significant level expression in the 30PRE treatment. ZMYND15 interacts with histone deacetylases and plays an essential role in the regulation of spatial-temporal expression of many haploid genes.

Moreover, is specifically expressed in spermatids during spermiogenesis process and is essential for normal spatiotemporal haploid gene expression. Male infertility and azoospermia have been linked to the inactivation of this gene in mice (Yan et al., 2010). In our study, ZMYND15 transcript presents low levels of relative expression in 30POST in comparison to the control. The expression pattern of this gene in invertebrates, mollusks or even cephalopods has not been described. This is the first report about the existence of this gene in cephalopods and its potential role in *O. maya* male infertility.

Another haploid gene required for male fertility during spermiogenesis is KLHL10. This gene is involved in protein ubiquitination. In mice is critical for the maturation process of spermatozoa, and is one of the essential proteins for post-meiotic spermatozoa (Yatsenko et al., 2010). KLHL10 is a member of a large BTB (Brica-brac, Tramtrack, and Broad-Complex)-kelch protein superfamily, characterized by an amino-terminal BTB/POZ domain and kelch repeats at the carboxyl terminus. This protein is specifically expressed in the testis and has similar expression pattern than CUL3. Wang et al. (2006) suggested that KLHL10 interacts with CUL3 to form a CUL3-based ubiquitin E3 ligase that functions specifically in the testis to mediate protein ubiquitination during spermiogenesis. This is the first time that KLHL10 is identified in mollusks and specifically cephalopods as *O. maya*. The RNA-Seq and qPCR analysis showed that KLHL10 presented the higher expression at 30°C in both PRE and POST condition. An increase in the expression values was observed directly proportional to the temperature. As was observed in human and mice, we can hypothesize that the increment in the expression of this transcript at 30°C affected the male fertility in *O. maya*. Disrupted spermatogenesis, degeneration of late spermatids and reduction in late spermatid number which was reported by López-Galindo et al. (2019) where they observed disruption in the germ cell strata of the seminiferous tubules and did not find a parental contribution from thermally-stressed fathers.

During the spermiogenesis process, haploid germ cells are transformed into highly polarized cells with the potential for motility and fertilization (Lo et al., 2012). The sperm tail, like motile cilia and flagella of other species, contains an axoneme at its core composed of a 9+2 microtubule arrangement. The axoneme develops from a single centriole at the base of the sperm head and functions to metabolize ATP and generate microtubule sliding and motility (Maxwell, 1974; Lo et al., 2012). Defects in sperm axoneme function result in asthenospermia (Abnormal sperm motility) (Lo et al., 2012). Lo et al. (2012) identified the RABL2 gene as critically involved in sperm tail function and male fertility in mice. In this study, RABL2 transcript, agree to RNA-Seq and qPCR analysis showed the highest expression in the 30PRE treatment. An expression increment from 24 to 30°C was observed. We hypothesize that the overexpression of this transcript starts with the response at 28°C, intending to repair the damage caused to the sperm tail. However, we can attribute that the reduced parental contribution and its lack at 30°C are directly related to the injury in sperm motility of *O. maya* males (López-Galindo et al., 2019).

Another gene involved in the sperm tail function is DNAJB13, which is a type II HSP40/DnaJ protein (Li and Liu, 2014). This gene is also known as testis spermatogenesis apoptosis-related protein expressed abundantly in mouse testis (Li and Liu, 2014). DNAJB13 was characterized in mature mouse testis and epididymal spermatozoa by Guan et al. (2010). Li and Liu (2014) confirmed the expression of DNAJB13 in the cytoplasm of spermatids and the flagella of mature spermatozoa, indicating its function in sperm motility. In our study, DNAJB13 transcript was also highly expressed at 30°C post copula.

According to the transcripts identified in response to thermal stress and spermatogenesis and spermiogenesis, it can be corroborated that the temperature significantly affects these processes carried out in the *O. maya* testis. These transcripts presented a high expression perhaps with the objective of compensating the damage caused by the rise in temperature. However, at the tissue level, these mechanisms are insufficient triggering inflammatory processes and tissue necrosis in the testis of thermally-stressed octopuses.

The increase in the expression of transcripts involved in spermatogenesis processes may explain the increase in the number of spermatophores observed in octopuses exposed to 28 and 30°C (López-Galindo et al., 2019). This expression patterns could demonstrate an effort at the reproductive level to compensate for the deleterious damage attributed to temperature.

The transcripts TSSK2, KLHL10, ZMYND15, RABL2A, and DNAJB13 indicated that there is a harmful effect on the production of viable sperm cells, the structural conformation of sperm, training, and motility. This was reinforced by histological analyzes, which show the moderate to severe damage to the testis and the lost of different cell types (spermatogonia, spermatocytes, spermatids, and mature spermatozoa) in *O. maya* males exposed to temperatures above 28°C. Chronic thermal stress generated infertility in *O. maya* which was corroborated through analysis of parental assignment, where no parental contribution of thermally-stressed parents was found.

## Conclusion

Under optimal temperature conditions, mating at the physiological, reproductive, and transcriptomic level does not represent a stressor for *O. maya* males. Under mating conditions, there is a significant expression of transcripts involved in the fundamental processes carried out in the testis such as spermatogenesis, gamete generation, and spermiogenesis. Under thermal stress, at the three levels mentioned above, there are severe alterations. The histology of the testis shows that temperature causes severe damage in the testis, affecting the morphology of the different cell types and the seminiferous tubules. These effects coupled with the transcriptomic analysis showed that there is a range of transcripts that are significantly expressed in response to thermal stress (response to stimuli, immune system, programmed cell death, apoptosis, regulation of cytokine production, necroptotic processes). At the reproductive level transcripts involved in spermatogenesis and spermiogenesis are significantly upregulated. When evaluating the combined effect of temperature and reproduction, at the transcriptomic level, there is a significant upregulation concerning optimal conditions. This pattern of expression reveals that under this condition, mating implies a stressor for the individual. In addition to the spermatogenesis processes in this condition, a large number of transcripts involved in energy production processes (catabolic and metabolic processes of amino acids, gluconeogenesis, glycolytic processes, fatty acid oxidation) were observed. This could indicate that in the testis of *O. maya* males a high amount of energy is produced for three essential aspects: to compensate the severe damage generated to the testis, to provide energy to be able to carry out the mating and try to produce more sperm. However, this strategy seems to be insufficient, since males of this species cannot produce offspring in spite of carrying out mating.

In the other hand, as observed in *O. maya* females and embryos, the problems presented a thermal threshold at 28°C, from which the physiological process, the reproductive performance and success, and the molecular mechanisms involved in the stress response and reproductive traits are severely affected. This study provides relevant information on the adaptive mechanisms presented in *O. maya* males against the effects of temperature. This is of vital importance, due to the predictions about the rise of sea temperatures between 2.5 and 3°C in the western zone of the Yucatan Peninsula causing a significant reduction of the population in this area, and/or migration to the eastern zone of the peninsula.

## Author Contributions

LL-G and CG-S conceived the project. LL-G performed the experiments and sample collection. LL-G and EL-S contributed to sample processing. LL-G and OJ conducted the bioinformatics analysis. LL-G, GDV, and CV-L conducted the qPCR validation and data analysis. CG-S, OJ, EL-S, and AL-L revised the manuscript.

## Conflict of Interest Statement

The authors declare that the research was conducted in the absence of any commercial or financial relationships that could be construed as a potential conflict of interest.

## Footnotes

1 http://www.bioinformatics.babraham.ac.uk/projects/fastqc/

2 http://www.ncbi.nlm.nih.gov/

3 https://github.com/trinityrnaseq/trinityrnaseq/wiki/Post-Transcriptome-Assembly-Downstream-Analyses
