# Supplementary material for: Transcriptomic Analysis Reveals Insights on Male Infertility in Octopus maya Under Chronic Thermal Stress
Source: Front Physiol. 2019 Jan 15;9:1920. doi: 10.3389/fphys.2018.01920 (PMC6341066; doi:10.3389/fphys.2018.01920)
Supplement: Supplementary file 1 [file Table_1.DOCX]

**Supplementary Table 1. Designed primers for selected DEGs used in qRT-PCR assays.** GC- Guanine-Citosine percentage; T - annealing temperature; e - Primer efficiency

| **Gen** | **RNA-seq Transcript** | **Primer Sequence (5’→3’)** | | **Position** | **Length** | **Fragment lenght (bp)** | **GC%** | **T (°C)** | ***e*** |
| --- | --- | --- | --- | --- | --- | --- | --- | --- | --- |
| *NFKB2* | TRINITY_DN11092_c0_g1_i1 | F | CTTCATGGCCAGTAGTACTTGC | 2435-2456 | 22 | 153 | 50.0 | 60 | 1.992 |
|  |  | R | AGAAAGAAGGCCCTGTCAAAAC | 2566-2587 | 22 |  | 45.5 |  |  |
| *ZMYND15* | TRINITY_DN17130_c0_g1_i2 | F | ATAATGAACTCCTCTCCGGGAC | 36-57 | 22 | 125 | 50.0 | 56 | 1.903 |
|  |  | R | ACATTCTCAACACGTCCGTTAC | 139-160 | 22 |  | 45.5 |  |  |
| *TDRD1* | TRINITY_DN9963_c0_g1_i1 | F | GTGAGGGTTGTCATCAGAATCG | 494-515 | 22 | 176 | 50.0 | 56 | 1.942 |
|  |  | R | GTTTGATTGATTTGGGTCACGC | 648-669 | 22 |  | 45.5 |  |  |
| *HTT* | TRINITY_DN2386_c0_g1_i1 | F | CGATTTCTTCCCACCTCAAGAC | 2652-2673 | 22 | 160 | 50.0 | 62 | 1.905 |
|  |  | R | CAACATAACCCAATCACGGACA | 2790-2811 | 22 |  | 45.5 |  |  |
| *MIF* | TRINITY_DN13245_c0_g1_i1 | F | GCCAATCTGCTTCGACATCATT | 314-335 | 22 | 165 | 45.5 | 60 | 1.99 |
|  |  | R | GCAGTTCTCTCCCTCGATTTTG | 457-478 | 22 |  | 50.0 |  |  |
| *GPX4* | TRINITY_DN26602_c0_g1_i1 | F | TTCCTCCGGTTCTGTTTGATTG | 29-50 | 22 | 155 | 45.5 | 60 | 1.909 |
|  |  | R | CCATCAATAGTCGTGGCAGAAA | 162-183 | 22 |  | 45.5 |  |  |
| *HSPA9* | TRINITY_DN33756_c0_g1_i1 | F | TTTATGATCTTGGCGGTGGAAC | 838-859 | 22 | 173 | 45.5 | 62 | 1.969 |
|  |  | R | GGACACATCTACTCCTTGGTCT | 989-1010 | 22 |  | 50.0 |  |  |
| *CASP7* | TRINITY_DN7707_c0_g1_i1 | F | ATTTGGCTGAGGTTCACGATTT | 672-693 | 22 | 151 | 40.9 | 60 | 1.991 |
|  |  | R | TCCTCGTTCGCATTAACTTTCC | 801-822 | 22 |  | 45.5 |  |  |
| *KLHL10* | TRINITY_DN16585_c0_g1_i1 | F | TCTTCCCCTCCTCTTTGCTATC | 2964-2985 | 22 | 124 | 50.0 | 60 | 1.939 |
|  |  | R | CAAATATTCCAAGGTCCCGGAC | 3066-3087 | 22 |  | 50.0 |  |  |
| *TSSK2* | TRINITY_DN17275_c0_g1_i1 | F | GCGCCGTACATTGTTCAAATAC | 3020-3041 | 22 | 158 | 45.5 | 60 | 1.968 |
|  |  | R | CGCCTGCCATATTGTGTAGAAT | 3156-3177 | 22 |  | 45.5 |  |  |
| *RABL2A* | TRINITY_DN7210_c0_g1_i1 | F | TGACCTGCAGTATCCCAGAAAT | 754-775 | 22 | 101 | 45.5 | 60 | 1.929 |
|  |  | R | AAACCACAACAATCATCCACGT | 833-854 | 22 |  | 40.9 |  |  |
| *CHD5* | TRINITY_DN16555_c1_g1_i2 | F | CAAACCTCTGCCACCTTTGTTA | 1937-1958 | 22 | 153 | 45.5 | 60 | 1.959 |
|  |  | R | AAATCTCGTACCAGCCATTGTG | 2068-2089 | 22 |  | 45.5 |  |  |
| *DNAJB13* | TRINITY_DN3007_c0_g1_i1 | F | CATTGAAAGACCATCCACACGA | 744-765 | 22 | 191 | 45.5 | 60 | 1.98 |
|  |  | R | TCCTTCTCCTGGCACTACTTTT | 913-934 | 22 |  | 45.5 |  |  |
| *TUFM* | TRINITY_DN35138_c0_g1_i1 | F | TTTTCCCCATAGATTCGGCTGT | 724-745 | 22 | 117 | 45.5 | 60 | 2.026 |
|  |  | R | AAGCACCGTGTCCCAGTATATC | 819-840 | 22 |  | 50.0 |  |  |
| *TUBGCP3* | TRINITY_DN14290_c0_g1_i1 | F | TTGATGGAGAGCAGCACAGAAA | 2043-2064 | 22 | 151 | 45.5 | 60 | 1.963 |
|  |  | R | GATCGGAATAGGGAGAGCAGTG | 2172-2193 | 22 |  | 54.6 |  |  |
