# Supplementary material for: Transcriptomic Analysis Reveals Insights on Male Infertility in Octopus maya Under Chronic Thermal Stress
Source: Front Physiol. 2019 Jan 15;9:1920. doi: 10.3389/fphys.2018.01920 (PMC6341066; doi:10.3389/fphys.2018.01920)
Supplement: Supplementary file 2 [file Table_2.DOCX]

**Supplementary Table 2. Search for key genes in stress response in testis of *O. maya* exposed to thermal stress and reproductive condition.** OSR – Oxidative stress response, PDC – Programmed death cell, RAP – Regulation of apoptotic process, RSR – Regulation of stress response, ROS – Response to oxidative stress, SR – stress response, IR – Inflammatory response. Asterisk indicates the treatment where the genes were significantly up-regulated. Proteins in bold indicate the good candidates to qPCR analysis.

| **Contig ID** | **UniProt ID** | **Protein Name** | **24POST** | | **30PRE** | **30POST** | **Biological process** | **E-value** |
| --- | --- | --- | --- | --- | --- | --- | --- | --- |
| TRINITY_DN17317_c0_g1_i3 | STK24_HUMAN | Serine/threonine-protein kinase 24 |  | |  | * | OSR | 6.02E-150 |
| TRINITY_DN7707_c0_g1_i1 | CASP7_HUMAN | **Caspase-7** |  | |  | * | PDC | 8.99E-25 |
| TRINITY_DN33756_c0_g1_i1 | GRP75_PONAB | **Stress-70 protein** |  | |  | * | RAP | 0.00E+00 |
| TRINITY_DN1469_c0_g2_i1 | ABR_MOUSE | Active breakpoint cluster region-related protein |  | | * | * | RSR | 2.11E-28 |
| TRINITY_DN15474_c0_g1_i1 | BABA1_DANRE | BRISC and BRCA1-A complex member 1 |  | | * | * | RSR | 4.81E-49 |
| TRINITY_DN12962_c0_g1_i1 | CO3_HUMAN | Complement C3 |  | | * | * | RSR | 4.98E-81 |
| TRINITY_DN641_c0_g1_i1 | PGBD3_HUMAN | PiggyBac transposable element-derived protein 3 |  | | * |  | RSR | 1.76E-29 |
| TRINITY_DN16710_c0_g1_i3 | PPM1B_RAT | Protein phosphatase 1B |  | | * | * | RSR | 6.94E-177 |
| TRINITY_DN17962_c9_g2_i3 | SETMR_HUMAN | Histone-lysine N-methyltransferase SETMAR |  | | * | * | RSR | 1.11E-40 |
| TRINITY_DN7225_c0_g2_i1 | SH3R1_XENTR | E3 ubiquitin-protein ligase SH3RF1 |  | |  | * | RSR | 1.54E-16 |
| TRINITY_DN17905_c8_g1_i1 | UBCD6_DROME | Ubiquitin-conjugating enzyme E2-17 kDa |  | |  | * | RSR | 4.42E-91 |
| TRINITY_DN15904_c0_g1_i1 | CSK_CHICK | Tyrosine-protein kinase CSK |  | |  | * | ROS | 0.00E+00 |
| TRINITY_DN26602_c0_g1_i1 | GPX4_BOVIN | **Phospholipid hydroperoxide glutathione peroxidase** |  | |  | * | ROS | 6.74E-72 |
| TRINITY_DN49654_c0_g1_i1 | KPYM_HUMAN | Pyruvate kinase PKM |  | |  | * | ROS | 7.93E-117 |
| TRINITY_DN10096_c0_g1_i2 | SODM_RAT | Superoxide dismutase [Mn] |  | |  | * | ROS | 7.07E-102 |
| TRINITY_DN11092_c0_g1_i1 | NFKB2_MOUSE | **Nuclear factor NF-kappa-B p100 subunit** |  | | * | * | SR/IR | 2.70E-100 |
| TRINITY_DN14073_c0_g1_i1 | C1QBP_BOVIN | Complement component 1 Q subcomponent-binding protein |  | |  | * | SR | 1.65E-24 |
| TRINITY_DN12030_c0_g1_i1 | CRIP1_HUMAN | Cysteine-rich protein 1 |  | |  | * | SR | 5.59E-29 |
| TRINITY_DN13057_c0_g1_i1 | DDX58_MOUSE | Probable ATP-dependent RNA helicase DDX58 |  | |  | * | SR | 3.25E-72 |
| TRINITY_DN11341_c0_g1_i1 | FLNA_HUMAN | Filamin-A |  | |  | * | SR | 0.00E+00 |
| TRINITY_DN50655_c0_g1_i1 | HSP7C_RAT | Heat shock cognate 71 kDa protein |  | |  | * | SR | 0.00E+00 |
| TRINITY_DN42859_c0_g1_i1 | ITA9_MOUSE | Integrin alpha-9 |  | |  | * | SR | 3.83E-49 |
| TRINITY_DN26506_c0_g1_i1 | MALT1_HUMAN | Mucosa-associated lymphoid tissue lymphoma translocation protein 1 | |  |  | * | SR | 4.19E-50 |
| TRINITY_DN13245_c0_g1_i1 | MIF_PIG | **Macrophage migration inhibitory factor** |  | |  | * | SR/IR | 1.18E-29 |
| TRINITY_DN16861_c0_g1_i4 | NAC3_HUMAN | Sodium/calcium exchanger 3 |  | |  | * | SR | 1.55E-66 |
| TRINITY_DN9614_c0_g1_i1 | PDCD6_HUMAN | Programmed cell death protein 6 |  | |  | * | SR | 9.47E-75 |
| TRINITY_DN8637_c0_g1_i1 | PLCG1_MOUSE | 1-phosphatidylinositol 4 |  | |  | * | SR | 1.70E-115 |
| TRINITY_DN18002_c2_g2_i5 | RIPK1_HUMAN | Receptor-interacting serine/threonine-protein kinase 1 |  | | * |  | SR | 1.53E-06 |
