# Supplementary material for: Transcriptomic Analysis Reveals Insights on Male Infertility in Octopus maya Under Chronic Thermal Stress
Source: Front Physiol. 2019 Jan 15;9:1920. doi: 10.3389/fphys.2018.01920 (PMC6341066; doi:10.3389/fphys.2018.01920)
Supplement: Supplementary file 3 [file Table_3.DOCX]

**Supplementary Table 3. Search for key genes in reproductive process and male fertility in testis of *O. maya* exposed to thermal stress and reproductive condition.** GG – Gamete generation, SG – Spermatogenesis, SpG – Spermiogenesis. Asterisk indicates the treatment where the genes were significantly up-regulated. Proteins in bold indicate the good candidates to qPCR analysis.

| **Contig ID** | **UniProt ID** | **Protein Name** | **24POST** | **30PRE** | **30POST** | **Biological process** | **E-value** |
| --- | --- | --- | --- | --- | --- | --- | --- |
| TRINITY_DN12132_c0_g1_i1 | KDM1B_MOUSE | Lysine-specific histone demethylase 1B |  |  | * | GG | 2.50E-151 |
| TRINITY_DN3082_c0_g2_i1 | MMP19_HUMAN | Matrix metalloproteinase-19 |  |  | * | GG | 9.53E-49 |
| TRINITY_DN3007_c0_g1_i1 | DJB13_MOUSE | **DnaJ homolog subfamily B member 13** |  |  | * | SG | 1.23E-122 |
| TRINITY_DN26459_c0_g1_i1 | AGM1_HUMAN | Phosphoacetylglucosamine mutase |  |  | * | SG | 0.00E+00 |
| TRINITY_DN7460_c0_g2_i1 | ASPM_FELCA | Abnormal spindle-like microcephaly-associated  protein homolog |  |  | * | SG | 2.56E-77 |
| TRINITY_DN16555_c1_g1_i2 | CHD5_MOUSE | **Chromodomain-helicase-DNA-binding protein 5** |  |  | * | SG | 0.00E+00 |
| TRINITY_DN2386_c0_g1_i1 | HD_MOUSE | **Huntingtin** |  |  | * | SG | 0.00E+00 |
| TRINITY_DN17881_c0_g1_i3 | PSME4_BOVIN | Proteasome activator complex subunit 4 | * | * |  | SG | 0.00E+00 |
| TRINITY_DN7210_c0_g1_i1 | RBL2A_PONAB | **Rab-like protein 2A** |  | * |  | SG | 5.95E-82 |
| TRINITY_DN9963_c0_g1_i1 | TDRD1_ORYLA | **Tudor domain-containing protein 1** |  | * | * | SG | 2.77E-13 |
| TRINITY_DN17275_c0_g1_i1 | TSSK2_MOUSE | **Testis-specific serine/threonine-protein kinase 2** | * | * |  | SG | 6.82E-47 |
| TRINITY_DN17766_c0_g1_i1 | ZAN_PIG | Zonadhesin |  | * |  | SG | 3.39E-50 |
| TRINITY_DN27478_c0_g1_i1 | ITB1_SHEEP | Integrin beta-1 |  |  | * | SG | 2.29E-128 |
| TRINITY_DN16585_c0_g1_i1 | KLH10_HUMAN | **Kelch-like protein 10** |  | * | * | SpG | 2.68E-142 |
| TRINITY_DN17130_c0_g1_i2 | ZMY15_HUMAN | **Zinc finger MYND domain-containing protein 15** |  | * |  | SpG | 3.03E-53 |
