# Supplementary material for: Transcriptomic Analysis Reveals Insights on Male Infertility in Octopus maya Under Chronic Thermal Stress
Source: Front Physiol. 2019 Jan 15;9:1920. doi: 10.3389/fphys.2018.01920 (PMC6341066; doi:10.3389/fphys.2018.01920)
Supplement: Supplementary file 5 [file Data_Sheet_1.PDF]

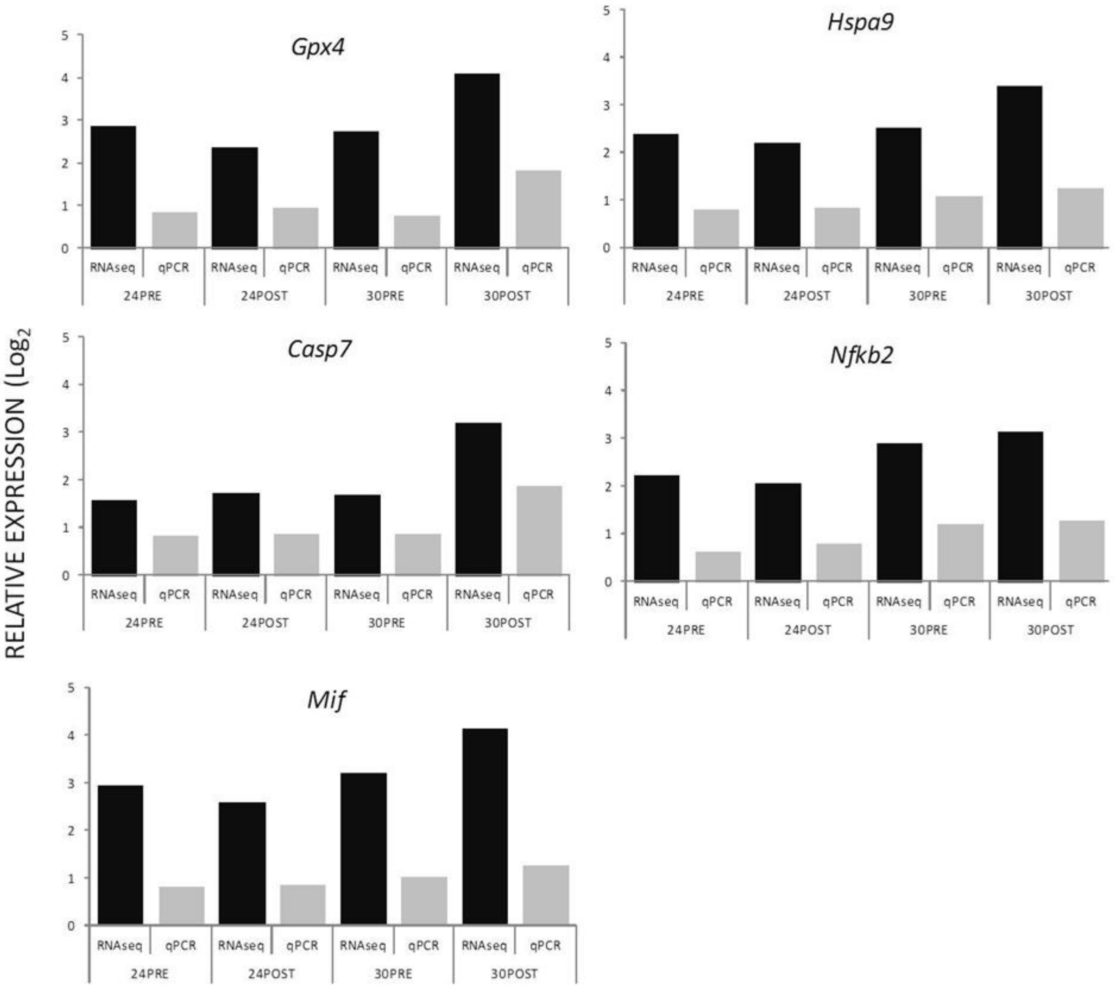

Supplementary Figure 1. RNA-seq and qPCR log_2 transformed expression for transcripts associated to thermal stress response.
